# Challenges in machine perfusion preservation for liver grafts from donation after circulatory death

**DOI:** 10.1186/2047-1440-2-19

**Published:** 2013-11-27

**Authors:** Naoto Matsuno, Eiji Kobayashi

**Affiliations:** 1 Department of Innovative and Transplant Surgery, National Center for Child Health and Development, 2-10-1 Okura, Setagaya, Tokyo 157-8535, Japan; 2 Tokyo Metropolitan University, 1-1 Minami-Osawa, Hachioji-shi, Tokyo 192-0397, Japan; 3 Center for Development of Advanced Medical Technology, Jichii Medical University, 3311-1 Yakushiji, Shimotsuke, Tochigi Prefecture 329-0498, Japan

**Keywords:** Liver machine perfusion, Liver transplantation, Donation after cardiac death

## Abstract

Donation after circulatory death (DCD) is a promising solution to the critical shortage of donor graft tissue. Maintaining organ viability after donation until transplantation is essential for optimal graft function and survival. To date, static cold storage is the most widely used form of preservation in clinical practice. However, ischemic damage present in DCD grafts jeopardizes organ viability during cold storage, and whether static cold storage is the most effective method to prevent deterioration of organ quality in the increasing numbers of organs from DCD is unknown. Here we describe the historical background of DCD liver grafts and a new preservation method for experimental and clinical transplantation. To prevent ischemia-reperfusion injury in DCD liver grafts, a hypothermic machine perfusion (HMP) technique has recently been developed and may be superior to static cold preservation. We present evidence supporting the need for improving liver perfusion performance and discuss how doing so will benefit liver transplantation recipients.

## Introduction

Approximately 100,000 patients worldwide undergo organ transplantation annually, but many other patients remain on waiting lists. The shortage of donors for transplantation is a universal problem. The waiting list has continued to grow, and the discrepancy between supply and demand is still increasing. The Declaration of Istanbul calls for a new paradigm of national self-sufficiency. As mandated in the Declaration, each country or region, guided by the ethical principles of the World Health Organization (WHO), should strive to provide a sufficient number of organs for its recipients from its own population [1]. In another strategy, the use of marginal donors is a promising way to increase the supply of graft tissue. In particular, the use of organs from non-heart-beating donors or donation after circulatory death (DCD) is increasing in importance as a potential source of vital organs for clinical transplantation. Maintaining organ viability after donation until transplantation is essential for optimal graft function and survival. The two current approaches to preservation prior to transplantation are simple cold storage (SCS) and machine perfusion (MP). SCS’s simplicity, low cost, and need for transport make it the method of choice at the majority of transplant centers. However, the major principle of simple hypothermic liver preservation is the reduction of metabolic activity. Although MP of the liver using hypothermia may have a theoretical advantage in providing metabolic support and oxygenation, its use has not become widespread in clinical practice. Recently, short- and long-term function of kidney procured from DCD by means of normothermic recirculation was reported [2]. The principle underlying normothermic and subnomothermic perfusion is recreation of the physiologic environment by maintaining normal organ temperature and providing essential substrates for cellular metabolism, oxygenation, and nutrition.

In this review, we summarize the historical background of liver transplantation from DCD, the subsequent development of clinical donor criteria for DCD liver grafts, and the progress of MP for DCD kidney and liver in cold storage are introduced. Finally, a promising preservation method of DCD liver transplantation is mentioned as a challenge using a new MP system. DCD liver transplantation can be performed for the many patients who desire the rescue from their death.

### Historical background of liver transplantation from DCD

Since the earliest days of liver transplantation, the critical shortage of donor liver grafts has promoted the creation of strategies to increase the donor pool. For example, a statement from the Institute of Medicine (IOM) and the Health Resources and Services Administration (HRSA) encouraged the use of grafts from high-risk donors in order to decrease the growing waiting list [3-5]. In March 1995, an international workshop on non-heart-beating donation was held in Maastricht, the Netherlands, and led to the Maastricht classification system (Table 1) for describing DCDs [6]. Categories 1, 2, and 4 include uncontrolled DCDs, and Category 3 includes controlled DCDs. DCDs have come to represent the fastest growing proportion of the donor pool. For example, in some United Network for Organ Sharing (UNOS) regions with limited standard criteria for donors, DCDs comprise 16% to 21% of the total donor pool [7]. Recent data show no difference between the long-term outcomes of transplantation of kidney grafts from DCDs and those from donation after brain death (DBD), although the incidence of delayed graft function (DGF) is higher for DCD kidneys [8-10]. Following this successful use of DCD kidney grafts for clinical transplantation, interest moved toward using extrarenal organs, including liver, pancreas, and lungs, from DCD [11]. Several transplant programs worldwide subsequently began to use livers from DCDs. However, early during these efforts, liver transplantations from DCDs did not show consistently favorable post-transplantation results. Livers from DCDs were found to frequently demonstrate diffuse hepatocytic necrosis, increased platelet adhesion, an absence of bile flow, and depletion of ATP. The development of ischemic biliary stricture was a major source of morbidity after DCD liver transplantation. Furthermore, although the incidence of DGF of transplanted kidneys was high, patients could receive hemodialysis until the kidneys recover. In contrast, DGF of early-phase liver transplants often required rescue therapy with retransplantation, which is associated with a significantly increased risk of patient mortality. This situation led to early marked reservations regarding the use of DCD liver grafts.

**Table 1:** Maastricht classification system

**Category**	**Features**	**Alternative categories**
1	Dead upon arrival, accident and emergency	Uncontrolled
2	Resuscitation attempted without success, accident and emergency	Uncontrolled
3	Awaiting cardiac death, intensive care	Controlled
4	Cardiac arrest while brain dead, intensive care	Controlled

Since the early days of DCD liver transplantation, the incidences of primary non-function and severe DGF have been reduced considerably due to the use of better selection criteria, livers from controlled DCD, and shorter warm and cold ischemic times. Unfortunately, even this improved strategy does not always significantly increase the numbers of potential donors. Additional studies are needed to identify clinical and policy strategies to reduce the incidence and improve the outcome of primary non-function and ischemic cholangiopathy in recipients of DCD liver grafts. One means to this end is to improve organ preservation methods and techniques.

### Donor criteria for DCD liver transplantation

Unlike the situation with kidney transplants, the use of livers from DCDs does not yet comprise the majority of transplants. There are no effective mathematical algorithms capable of differentiating livers on the basis of transplantability. Several recent large-scale studies on risk factors associated with DCD liver transplantation are summarized in Table 2. In one study, Mateo *et al*. analyzed the UNOS database of 367 liver transplants from DCDs and 33,111 liver transplants from DBD performed between 1996 and 2003 [12]. The graft survival rates at 1 year and 3 years in the recipients of livers from DCD donors (71% and 60%, respectively) were significantly (P <0.001) inferior to those with grafts from DBD donors (80% and 72%, respectively). The authors identified the following cumulative relative risk factors for graft loss among recipients: being on life-support, being hospitalized or placed in an intensive care unit, receiving dialysis, having a serum creatinine level greater than 2.0 mg/dL at the time of transplantation, and age greater than 60 years. However, 1-year and 3-year graft survival rates (81% and 67%, respectively) in low-risk recipients with low-risk DCD livers (that is, donor warm ischemic time (DWIT) of less than 30 minutes and cold ischemic time (CIT) of less than 10 hours) were not significantly different from those of recipients with DBD livers.

**Table 2:** Risk factors associated with DCD liver transplantation

**Risk factors**	**References**
Life support in ICU, dialysis, serum creatinine >2 mg/d, donor age >60 years, DWIT ≥30 minutes, CIT >10 hours, retransplantation	Mateo et al. [12]
Donor age >45 years, DWIT >15 minutes, CIT >10 hours	Lee et al. [13]
Donor age ≥55 years, male, African-American, HCV+, metabolic disorder, MELD ≥35, life support	Muthur et al. [14]
DWIT ≥30 minutes, MELD >30, donor age >60 years, CIT ≥10 hours	de Vere et al. [15]

CIT, cold ischemic time; DCD, donation after circulatory death; DWIT, donor warm ischemic time; HCV, hepatitis C virus; MELD, Model for End-stage Liver Disease.

In another study, Lee *et al*. used a multivariate Cox model to analyze data from 874 adult DCD liver transplantations performed between 1996 and 2006 [13]. Five risk factors were identified according to index scores: medical history, life support status at transplantation, DWIT, CIT, and donor age. Specifically, graft survival in recipients with low-risk DCD donors (age ≤45 years; DWIT ≤15 minutes; CIT ≤10 hours) was comparable to that of recipients with livers from DBDs. Mathur and coworkers analyzed data from the Scientific Registry of Transplant Recipients (SRTR) for DCD liver recipients who underwent transplantation between 1 September 2001 and 30 April 2009 (*n* = 1567) [14]. Recipient factors significantly (P ≤0.05) predictive of graft failure included: age 55 years or older; male; African-American; positivity for hepatitis C virus (HCV); presence of metabolic liver disorders; transplant Model for End-stage Liver Disease (MELD) score of 35 or greater; hospitalization at transplantation; and the need for life support at transplantation. Donor characteristics predictive (P ≤0.005) of graft failure included age 50 years or older and weight greater than 100 kg. Each additional hour of CIT was found to be associated with a 6% increased graft failure rate (P <0.001), and DWIT of 35 minutes or more significantly (P = 0.002) increased the rate of graft failure. Factors predictive (P ≤0.006) of recipient mortality included age 55 years or older, hospitalization at transplantation, and retransplantation. The Pittsburgh group identified DWIT as the most indicative parameter. Specifically, they found that DWIT exceeding 20 minutes is associated with significantly poorer graft survival rates. Surprisingly, CIT is not similarly associated with poor outcomes in recipients of DCD transplants. However, DCD and DBD grafts transplanted into patients whose MELD score exceeded 30 or who were on organ-perfusion support (mechanical ventilation or hemodialysis) had similar survival rates, suggesting a potentially greater benefit of DCD livers in critically ill patients [15]. Furthermore, patients who had undergone liver transplantation from DCD donors who were older than 60 years had a markedly high rate (67%) of biliary complications. Factors associated with ischemic cholangiopathy, including older donor age, high donor weight, CIT, and DWIT, were significant predictors of graft failure. Currently, general criteria for using DCD livers for transplantation include DWIT of less than 30 minutes, donor age of 60 years or less, and CIT of no more than 8 to 10 hours (Table 2).

### MP preservation of grafts (kidney and liver) during cold storage

The introduction of kidney perfusion preservation in clinical practice started in the late 1960s and built on then-recent advances in continuous hypothermic isolated perfusion using blood [16] and cryoprecipitated plasma prior to autologous kidney transplantation. Subsequently, dextrose, insulin, hydrocortisone, penicillin, and magnesium sulfate were added to the plasma [17]. One of the most noteworthy achievements followed in 1967, in which canine kidneys were transplanted successfully after 72 hours of pulsatile hypothermic MP preservation [18]. Hypothermic machine perfusion (HMP) of human kidney became a clinical reality soon thereafter: a patient who received a kidney that had been preserved for 17 hours by using this preservation circuit achieved acceptable post-transplantation function [19]. During the 1970s, HMP was used by transplant centers (mainly in the United States and Europe) to preserve and transport kidneys. In addition, several groups improved the existing perfusion solution by adding or omitting various components. Consequently, different perfusion machines were developed and used clinically for kidney preservation. For example, the equipment used during the 1970s by Koostra and colleagues was a modified Gambro machine [20]. In comparison, the Newcastle DCD kidney team used similar equipment but a locally manufactured UW-like solution that lacked starch [21]. However, the development of UW solution in 1980 allowed surgeons to preserve kidneys by SCS for much longer times (maximum 72 hours) than had been possible previously [22]. Combined with SCS, the UW solution provided a cost-effective alternative to MP, and most centers abandoned the clinical use of MP soon thereafter. Over the last few decades, the success of kidney transplantation as the treatment of choice for end-stage renal failure has led to an increasing shortage of suitable organs. This shortage has forced the transplantation community to reconsider the use of grafts from otherwise high-risk donors, including aged or hemodynamically unstable donors, and those from non-heart-beating donation. Consequently, MP of kidneys from these marginal donors regained worldwide interest. MP preservation methods that provide an optimized physiologic environment that enables organ evaluation, resuscitation, and modulation before transplantation are now the focus of research and clinical use.

The international multicenter trial for HMP in kidney transplantation is a well-designed prospective randomized trial of paired kidneys [23]: one was preserved by using HMP and the other by using SCS. The study examined 672 renal transplantations performed in Europe. HMP significantly reduced the duration of DGF and improved the rate at which the serum creatinine level decreased. In addition, the risk of DGF was significantly reduced by HMP compared with cold storage (26.5% versus 20.8%; P = 0.05), and in recipients who did develop DGF, the 6-month graft survival rate was higher when kidneys were machine-perfused than cold-stored (87% versus 76%; P = 0.05). Furthermore, the 1-year graft survival rate improved from 90% in the SCS group to 94% for HMP kidneys. As part of this clinical trial, Treckmann *et al*. analyzed the possible effects of MP versus cold storage on DGF and early graft survival in expanded-criteria donors [24]. The technology and utilization of HMP have increased exponentially with time, and now 25% to 35% of all kidneys transplanted in the United States are preserved by using HMP [25]. In addition, pre-transplantation viability tests of DCD grafts are particularly important. Making a reliable assessment of DCD grafts is difficult for several multifactorial reasons, and performing liver biopsies is of limited value, even in brain-dead potential donors. One advantage of MP preservation is that viability tests can be performed on grafts while they are stored. To this end, a clinical study used trypan blue exclusion with collagenase digestion of biopsy tissue to demonstrate hepatocyte viability in DCD liver grafts for transplantation [26]. In addition, HMP enables surgeons to judge the suitability of a graft by assessing organ flow and pressure characteristics and by analyzing various enzyme levels in the perfusate. Developing an MP system to support viability assessments of liver grafts has been complicated by their unique blood supply. Predicting viability by evaluating flow in the portal system has been problematic because portal flow is wide-ranged and early MP systems were unable to generate sufficient portal pressure to yield adequate portal flow during the hypothermic stage of preservation. Even tissue and vascular resistance, which provide important information in kidney preservation, are particularly low in liver due to easy destruction. However, previous reports have shown that the levels of aspartate aminotransferase (AST) and lactate dehydrogenase (LDH) in circulating preservation solution are useful and predictable biomarkers of liver graft health [27-29]. In addition, Obara *et al*. recently developed a novel liver MP system and found that the degree of decrease in hepatic arterial pressure is significantly correlated with the duration of warm ischemic injury and with the levels of liver enzymes (AST, LDH) in cold perfusate during continuous preservation [29].

Advantages and disadvantages of MP preservation are summarized in Table 3.

**Table 3:** Advantages and disadvantages of machine perfusion (MP) preservation

**Advantages**	**Disadvantages**
Lower incidence of delayed graft function	Higher cost in the short-term
Continuous monitoring of parameters	Endothelial injury is possible
Decrease vasospasm	Logistically more complex
Ability to provide metabolic support	Possible equipment failure
Potential for pharmacologic manipulation	

### Current challenges in applying new machine preservation methods to DCD liver grafts

Despite the example of the successful application of MP to DCD kidney grafts, the application of HMP to DCD liver transplantation has remained challenging. The optimal parameters for the use of HMP in DCD livers have not been established. *Ex vivo* assessment of liver damage showed that 10 hours of HMP reduced the cellular damage induced due to 30 minutes of DWIT [30]. Dutkowski and colleagues studied the effect of short-term hypothermic oxygenated perfusion (HOPE) at the end of cold storage and found that 1 hour of HMP after 45 minutes of DWIT and 5 hours of SCS improved the status of rat liver grafts, with a reduction in hepatocyte necrosis, less AST release, and increased bile flow [31]. Various other studies have investigated the effects of changing the perfusion temperature. For example, subnormothermic MP at 20°C decreased vasoconstriction and metabolic requirements in DCD [32] and steatotic [33] rat models. However, the limitations of basic transplantation research using small animal models include difficulties in the assessment of hepatic artery flow. These limitations are important factors when weighing the clinical relevance of small animal preservation studies. In large animal studies, successful liver transplantation in a canine model was achieved after 24 hours of MP [34]. The first 11 human livers to be transplanted were perfused for as long as 7.5 hours by using the same method as in the previous canine study [35]. However, the use of fresh diluted blood is inconvenient in the clinical setting. Pienaar *et al*. reported that canine livers could be preserved successfully for 72 hours by HMP via the portal vein alone [36]; no other similar results in large animal transplantation models have been published so far. Low pressure HMP was applied to porcine livers via the hepatic artery for 2 hours before transplantation; these HMP-treated grafts were then compared with similar grafts that had been stored in cold Euro-Collins solution for the same duration. LDH and AST levels were consistently lower in the HMP grafts compared with the SCS livers [37]. Guarrera and coworkers demonstrated the outcomes of liver transplantation in a miniature swine model after grafts had been exposed to 12 hours of SCS compared with 12 hours of HMP using Vasosol solution, a modified Belzer’s MP solution [38]. Serum AST and total bilirubin levels were similar between the HMP and SCS livers, indicating that HMP with a synthetic perfusate can be used successfully to preserve donor livers before transplantation. Applying this same solution and technology to human livers achieved satisfactory transplantation outcomes compared with those after SCS [39]. The MP preservation solution Polysol was developed in 2005 and contains many vitamins and a protein-like, enriched tissue culture medium for functional recovery during preservation, which is expensive [40,41]. Regarding DCD liver grafts in large animals, most groups had agreed that 30 minutes of DWIT plus 4 to 5 hours of cold preservation results in primary loss of function in pig liver [42,43]. In response, de Rougemont and colleagues tested whether HOPE-treated DCD pig livers experienced the same benefits as those noted in a previous report using a rat model [44]. The study showed that DCD porcine liver with 60 minutes of DWIT and preserved for 6 hours with SCS could be rescued by short-term (1 hour) HOPE [44]. Specifically, HOPE-treated pig livers had lower values of AST and LDH after reperfusion and a higher survival rate of up to 30 hours. Considering the potential benefit of temperature on liver preservation, Monbaliu and colleagues designed a multifactorial biological modulation approach targeting ischemia-reperfusion injury to augment the viability of porcine liver grafts with 45 minutes of DWIT and 4 hours of SCS [45]. In the modulation group, DCD livers were flushed with warm Ringer’s solution containing streptokinase and a vasodilator prior to SCS; recipients received glycine, a mitogen activated protein (MAP) kinase inhibitor, α-tocopherol, glutathione, and an iron chelator (apotransferrin) intravenously. This approach was effective and eliminated primary non-function, thus improving graft function [45].

**Figure 1:**
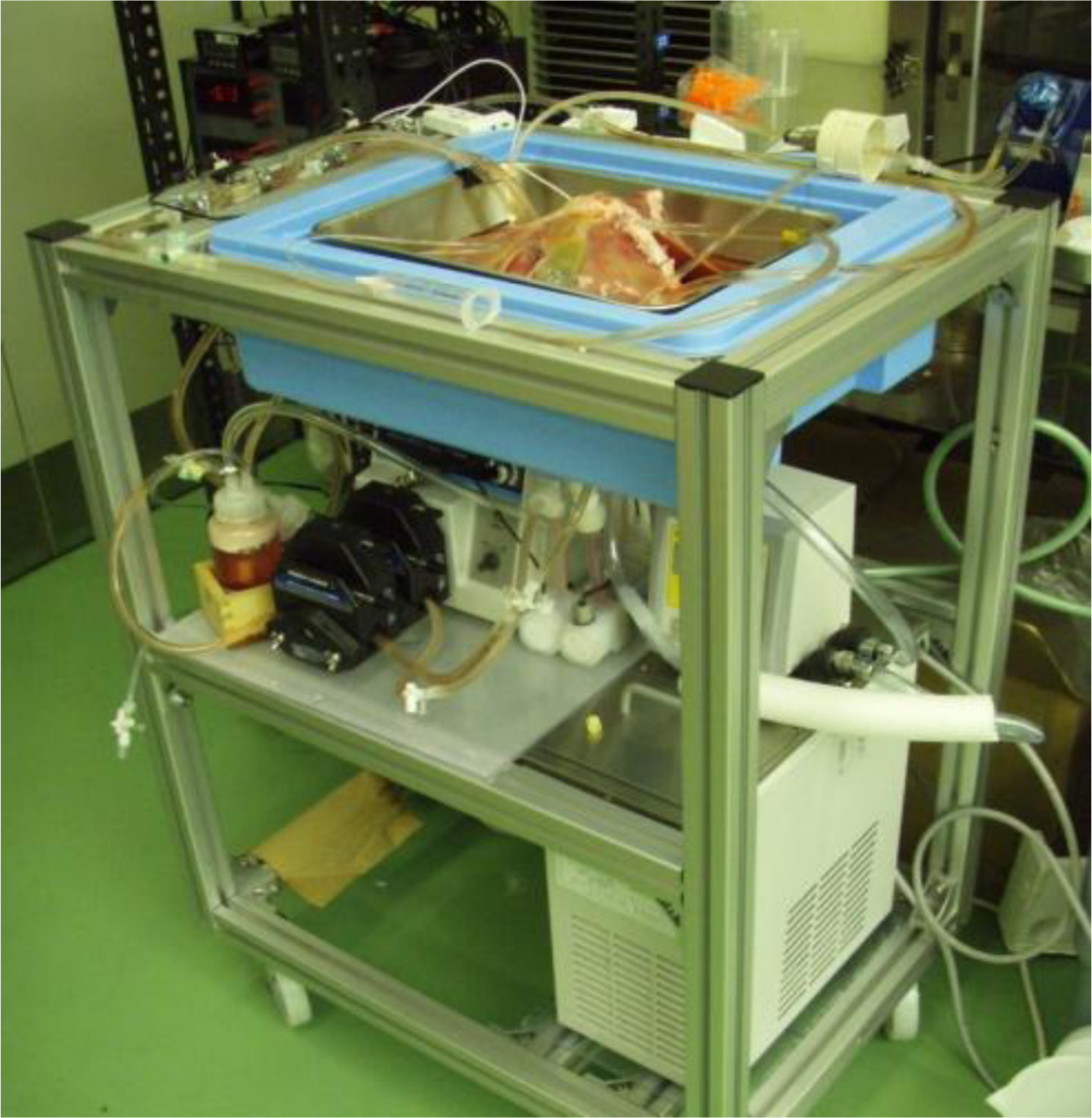
Temperature-controlled preservation machine.

In another temperature-associated strategy, we have developed a temperature-controlled preservation machine (Figure 1) and demonstrated beneficial functional recovery in a porcine liver transplantation model after 30 minutes of DWIT plus 4 to 5 hours of total ischemic time in the HMP group compared with the SCS-only group [46]. Furthermore, we successfully transplanted porcine livers that had been exposed to 60 minutes of DWIT plus 4 hours of total ischemic time by rewarming them from 4°C to 22°C during preservation using MP [47]. The traditional understanding and procedures regarding DCD grafts tend to be changed. Experimental studies have demonstrated that even brief periods of cold preservation injures hepatocytes, Kupffer cells, and endothelial cells in DCD livers, even in grafts that later are recirculated under normothermia [48-50]. The use of normothermic extracorporeal membrane oxygenated (NECMO) perfusion is based on experimental studies, which have shown that the recirculation of oxygenated blood at 37°C improves the cellular energy load, reduces tissue injury, and improves the post-transplantation graft function in livers damaged during the warm ischemia caused by cardiac arrest [51,52]. In 2002, the Hospital Clinic, University of Barcelona, Barcelona, Spain, developed a clinical protocol for resuscitating donor organs and maintaining their viability for transplantation [53]. The protocol includes cannulation of the donor’s femoral vessels to establish a NECMO circuit. NECMO is then used to reperfuse and deoxygenate abdominal organs after cardiac arrest while the potential donor is evaluated and consent for organ donation is obtained. In addition, using NECMO to maintain organs offers the theoretical benefit of applying cytoprotective substances that can support functional recovery.

**Figure 2:**
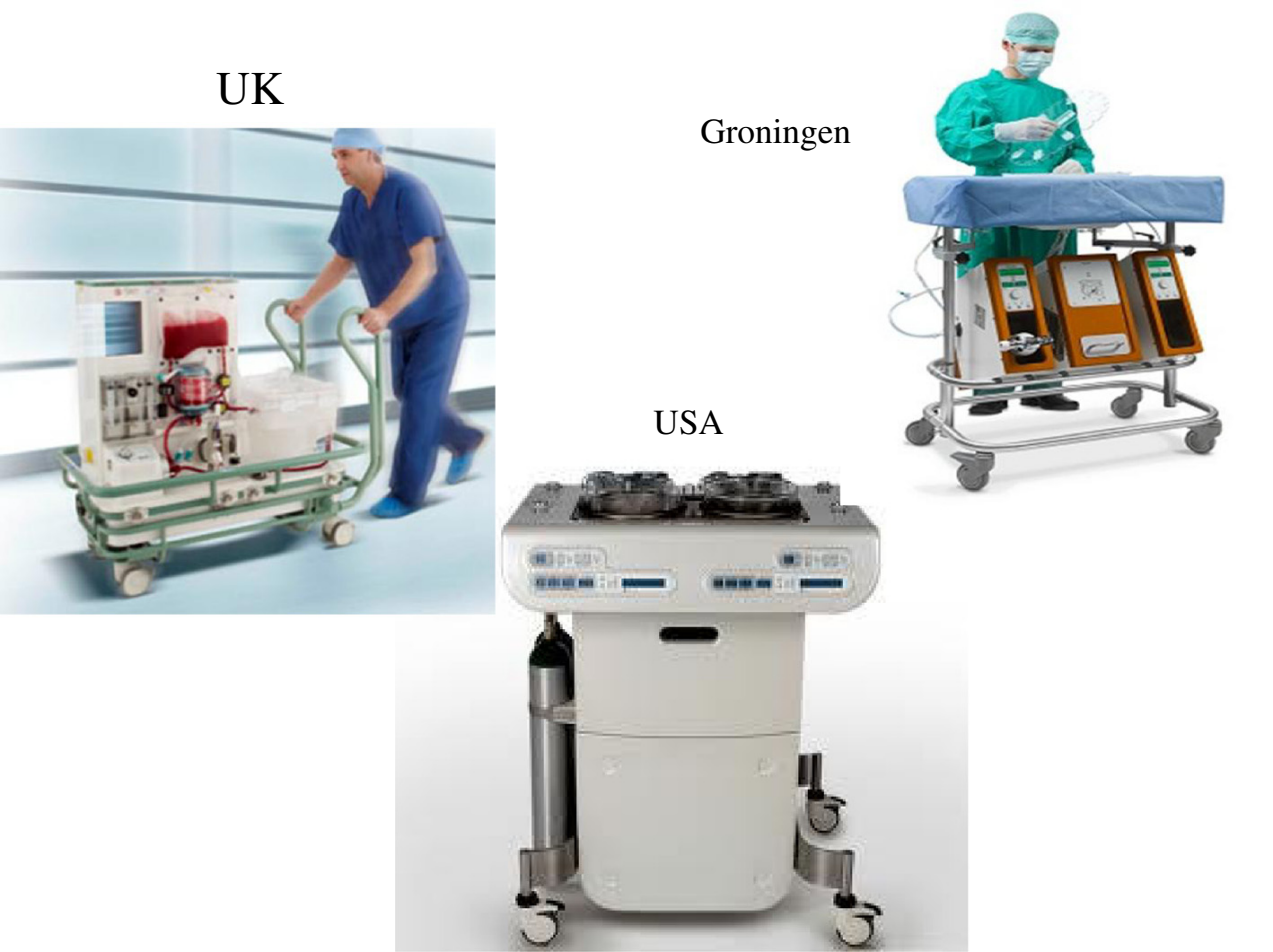
**Machine preservation systems used worldwide.** The perfusion preservation machine in the UK is applicable for both cold and normothermic conditions (left). The machine developed in the USA is for cold preservation (bottom).

During 2007, the first human liver transplantations were performed with livers from uncontrolled DCDs during which the donor was maintained with NECMO prior to organ retrieval. Ten DCD livers were transplanted, with only one graft lost to primary nonfunction and another to hepatic artery thrombosis. The DCD experience of the Barcelona group is continuing to increase with more than 40 cases, which however need much more donor cannulations for *in situ* NECMO [54]. A great advantage of normothermic preservation is the ability to overcome the deleterious aspects of hypothermic cellular physiology; however, the logistics of clinical organ retrieval might necessitate a period of cold preservation during transportation between institutions. In addition, the use of blood-based perfusates may increase the risks of microvascular failure, sinusoidal plugging, and bacterial growth [55]. Also, any equipment failure results in unexpected warm ischemic injury. Achieving normothermic liver preservation therefore remains challenging, but progress is being made: in March 2013, the BBC reported two human livers maintained with warm perfusion were successfully transplanted at King’s College Hospital, London, UK. Figure 2 shows various MP systems that are currently being used worldwide.

## Conclusions

This historical review of DCD liver preservation for clinical use has revealed that traditional methods of preservation based on hypothermic static storage are likely suboptimal for DCD liver grafts, because liver organs from DCDs have already suffered severe tissue damage secondary to hypoxia and hypoperfusion before the initial period of warm ischemia. Additional damage to the organ due to hypothermic conditions may limit the ability to restore cellular function because metabolic activity is decreased at low temperatures. However, at present it is unclear whether DCD livers can be better rescued by normothermic or hypothermic approaches. Ideally, DCD livers should be perfused continuously with oxygenated perfusate during the entire *ex vivo* phase of preservation. Moreover, preservation schemes should be established to overcome severe reperfusion injury, particularly ischemic cholangiopathy, in DCD livers. Finally, we propose using the term ‘organ regeneration’ to describe the *ex vivo* resuscitation of a marginal donor organ.

## Abbreviations

AST: Aspartate aminotransferase; ATP: Adenosine triphosphate; CIT: Cold ischemic time; DBD: Donation after brain death; DCD: Donation after circulatory death; DGF: Delayed graft function; DWIT: Donor warm ischemic time; HCV: Hepatitis C virus; HMP: Hypothermic machine perfusion; HOPE: Hypothermic oxygenated perfusion; HRSA: Health Resources and Services Administration; IOM: Institute of Medicine; LDH: Lactate dehydrogenase; MAP: Mitogen activated protein; MELD: Model for End-stage Liver Disease; MP: Machine perfusion; NECMO: Normothermic extracorporeal membrane oxygenated; SCS: Simple cold storage; SRTR: Scientific Registry of Transplant Recipients; UNOS: United Network for Organ Sharing; WHO: World Health Organization.

## Competing interests

EK has been a visiting professor at CDAMTec and a special advisor to Otsuka Pharmaceutical Factory Inc (Naruto, Japan) since 2009.

## Authors’ contributions

MN and EK designed and coordinated all of the projects. Both authors read and approved the final manuscript.
